# Development of Functional Pizza Base Enriched with Jujube (*Ziziphus jujuba*) Powder

**DOI:** 10.3390/foods11101458

**Published:** 2022-05-18

**Authors:** Aniello Falciano, Angela Sorrentino, Paolo Masi, Prospero Di Pierro

**Affiliations:** 1 Department of Agricultural Sciences, University of Naples Federico II, Portici, 80055 Naples, Italy; aniello.falciano@unina.it (A.F.); paolo.masi@unina.it (P.M.); prospero.dipierro@unina.it (P.D.P.); 2 Centre for Food Innovation and Development in the Food Industry, University of Naples Federico II, Portici, 80055 Naples, Italy

**Keywords:** pizza base, jujube fruit, functional food, antioxidant activity, polyphenolic compounds

## Abstract

Functional and enriched foods are increasingly in demand in the global market due to their benefits for human health and their prevention of several diseases. The aim of this work was to develop a functional pizza base, produced in the Neapolitan style, exploiting the beneficial properties of jujube. The jujube fruit is rich in phenolic compounds with high antioxidant activity and represents a good candidate for functional food development. The doughs were prepared by replacing the wheat flour with 2.5%, 5.0%, and 7.5% (*w*/*w*) of Ziziphus jujube powder (ZJP) and were subsequently cooked. Chemical analyses showed that both total phenolic compounds and antioxidant activity grew with the increase of ZJP. The addition of ZJP darkened the pizza base and raised its hardness, gumminess, and chewiness. However, no difference was found in the springiness and cohesiveness of the samples with or without ZJP. These results suggest that jujube powder can be successfully introduced into pizza dough as a functional ingredient.

## 1. Introduction

In recent years, a growing demand for food products with functional properties has been observed. Those foods, known as functional foods, are classified as fortified, enriched, or enhanced foods. Phytochemicals and phenolic antioxidants in plants, including fruits, vegetables, herbs, and spices, are recognized as active ingredients to be used in functional food. Among food products, baked goods are considered as consumer products, so the current trend in the baked goods industry is to create baked goods with health-beneficial attributes. The use of composite flour (a blend of wheat and non-wheat flours) may provide additional nutrients contained in the non-wheat material, thus improving the nutritional value of the bakery products [1]. Hence, in relation to good health demands, the nutritional value of wheat-based food products can be enhanced by supplementation with other nutrients from different sources [2].

There are many studies available on the development of functional bakery foods such as bread [3,4,5], cookies [6], biscuits [7], and cakes [8]. 

Among bakery products, pizza is consumed and liked throughout the world. Due to the simplicity of its preparation and good taste, pizza is also a popular snack that could be a promising vehicle for functional compounds, thus satisfying health-conscious customers [9,10]. Vitamins, minerals, dietary fibers, and phytochemicals present in plants contribute to the functionality of foods that they enrich. However, to satisfy consumers, it should not be overlooked that the addition of functional compounds must preserve or improve the sensory characteristics of the final products.

The jujube plant (*Ziziphus jujuba*, Mill) belongs to the *Rhamnaceae* family, and it is largely diffused in China. Nowadays, its cultivation is also found in other regions of the world, including Russia, South Asia, Southwestern United States, Australia, and Southern Europe. The fresh jujube fruit and its derivatives (paste, puree, syrup, etc.) have been largely used in traditional Chinese medicine, and as a dietary supplement with high amounts of bioactive compounds such as dietary fibers, mineral, and natural antioxidant compounds, such as phenols and flavonoids. It is well-known that the presence of phenolic compounds in food can be particularly important for consumers, both for their antioxidant properties and other biochemical properties which prevent the development of diseases such as neurodegenerative diseases [11]. Its interesting phytochemical composition makes the jujube a good candidate for exhibiting beneficial activity against various pathologies. In fact, in addition to its antioxidant capacity, numerous pharmacological properties have been identified; for example, its contribution to the prevention of cancer, and its anti-inflammatory, anti-obesity, immunostimulant as well as gastro- and hepato-protective activity [12]. Nevertheless, due to the short shelf-life of the fresh product, jujube powder was recently proposed as the best product to be used in many food formulations for the development of functional foods [13].

In this context, the present study aims to exploit the beneficial properties of jujube powder by using it to make composite flours in the development of a functional pizza base, produced in the Neapolitan style. The total phenol and antioxidant properties of pizza base containing ZJP were analyzed after baking and compared with the control. In addition, the texture attributes and the chromatic analysis results of the samples were also evaluated.

## 2. Materials and Methods

### 2.1. Chemicals

Methanol, Folin–Ciocalteu’s (FC) reagent, gallic acid, aluminum chloride, potassium acetate, DPPH (2,2-diphenyl-1-picrylhydrazyl), ABTS (2,2′-azinobis-3-ethylbenzothiazoline-6-sulfonic acid), and other chemicals were procured from Carlo Erba (Milano, Italy). The *Ziziphus jujuba* fruits (Meimizao variety) were provided by the arboriculture section of the Department of Agricultural Sciences, University of Naples Federico II, Portici, Naples, Italy.

### 2.2. Ziziphus jujuba Powder (ZJP) Preparation

The intact, ripened jujube fruits were washed with distilled water to remove impurities and then pitted. The pitted fruits were stratified on perforated trays and dried under a stream of hot air (2 m/s) at 40 °C for 72 h. The dried samples were ground using a laboratory mill (Model 3100, Perten Instruments Italia Srl, Rome, Italy) with a 0.5 mm sieve. The obtained powder was further sieved at 0.2 mm to obtain a homogeneous particle size. The ZJP obtained was packaged in a hermetically sealed, dark glass jar and stored at room temperature until use.

### 2.3. Chemical Analysis of ZJP

The soluble (SDF) and insoluble (IDF) dietary fiber contents were determined according to the gravimetric enzymatic method, as previously described by Prosky et al. [14]. Protein content (N × 6.25) and total fat were measured using Kjeldahl’s method and the Soxhlet apparatus, respectively. Total carbohydrates were evaluated by the phenol sulphuric acid method [15]. Moisture content was assessed using the AOAC method [16]. Ash content was detected by keeping the sample (3 g) at 550 °C in a muffle furnace for 5 h.

### 2.4. Preparation of the Pizza Base

The dough was prepared in the Neapolitan way. The recipe included 60% soft wheat flour type “00” (Caputo Rossa Pizzeria; 74% total carbohydrates, 13% protein, 1.5% fat, and 0.02% ash) (Antimo Caputo S.r.l., Napoli, Italy), 38% deionized water, 1.9% sodium chloride from Sicily (Italkaly, Palermo, Italy), and 0.1% fresh yeast (Lievital, Lesaffre Italia S.p.a, Parma, Italy). For the preparation of the functional pizza base, the wheat flour was replaced with 2.5% (ZJP-2.5), 5% (ZJP-5), and 7.5% (ZJP-7.5) (*w*/*w*) ZJP. The ingredients were mixed using the spiral mixer (Grilletta IM5, Famag S.r.l., Milano, Italy) for 18 min, and then 250 g loaves were formed and leavened in a climatic cell (Binder, type KBF-S, Tuttlingen, Germany) at 22 °C and 80% relative humidity for 16 h. Finally, the loaves were rolled and baked for 90 s (floor: 400 °C; vault: 450 °C) in an electric oven (iDeck, iD60/60D, Moretti Forni S.p.A., Pesaro and Urbino, Italy) with a refractory stone on the floor of the oven. The cooked samples were allowed to cool at room temperature before use. For chemical analyses, whole pizzas were cut into small pieces, freeze-dried, ground, sieved though a 0.2 mm sieve, and stored at −20 °C.

### 2.5. Preparation of Methanolic Extracts for Analysis

ZJP or pizza base powder (1 g) were mixed with 25 mL of aqueous methanol (70% *v*/*v*) and swirled at room temperature for 2 h. The samples were then centrifuged at 12,000× *g* for 15 min in a centrifuge at 20 °C. The supernatants were recovered and stored on ice in the dark, and the pellets were subjected to another extraction. Finally, the supernatants were collected and stored at −23 °C until the analysis.

### 2.6. Total Phenol and Flavonoid Content

The total phenolic content (TPC) was determined according to Sun et al. [17], with slight modifications. The extracts (50 μL) were mixed with 70 μL FC reagent and 880 μL distilled water. The mixture was thoroughly mixed by vortex for 1 min and incubated for 5 min at room temperature. Subsequently, 530 μL distilled water and 70 μL of 7.5% (*w*/*v*) sodium carbonate were added to each tube and incubated at 45 °C in the dark for 15 min; then, the absorbance was measured at 760 nm using the UV-VIS spectrophotometer (V-730, JASCO International Co Ltd., Sennincho Hachioji, Japan). Gallic acid was used as standard, and the results were expressed as mg of Gallic Acid Equivalent (GAE)/g dry weight (DW). Total flavonoid content (TFC) was measured according to Sagar and Pareek [18], without modifications. The extracts (0.5 mL) were poured into the tubes containing 1.5 mL methanol (80%) and subsequently mixed. Then, 1 M potassium acetate (0.1 mL), 10% aluminum chloride (0.1 mL), and distilled water (2.8 mL) were added, mixed, and incubated at room temperature for 30 min. After incubation, the absorbance was measured at 410 nm. The standard used was quercetin, and the results were expressed as mg quercetin equivalent (QE)/g DW.

### 2.7. Antioxidant Activity

The antioxidant activity was detected by using both the ABTS^•+^ and DPPH^•^ assays according to the method of Duan et al. [19]. Briefly, freshly prepared ABTS solution (7 mM) was incubated with 2.45 mM potassium persulfate (final concentration) in the dark at room temperature for 12–16 h to obtain the cationic radical (ABTS^•+^) before use. For the analyses, the ABTS^•+^ solution was diluted in 96% ethanol to an absorbance of 0.7 (±0.02) at 732 nm, then 1 mL of this solution was mixed with 25 μL of 70% methanol (blank) or sample extracts. The samples were incubated for 10 min at room temperature, and then the absorbance at 732 nm was measured.

The methanolic solution of DPPH^•^ (0.1 mM) was freshly prepared, and then 950 μL was mixed with 50 μL of the sample extract or 50 μL of methanol (blank). The samples were incubated in the dark at room temperature for 1 h, and then the absorbance at 517 nm was measured.

Radical scavenging activity was calculated using the following Formula (1):

ABTS^•+^ or DPPH^•^ scavenging activity (%) = (A_b_ − A_s_)/A_b_ × 100, (1)

where A_b_ = absorbance of the blank sample, and A_s_ = absorbance of the extract.

### 2.8. Texture Profile Analysis (TPA) of Cooked Pizza Base

Textural properties, including hardness, chewiness, cohesiveness, springiness, adhesiveness, and gumminess, were investigated by using a texture profile analyzer (TMS-Pro Texture Analyzer, Food Technology Corporation, Sterling, VA, USA). Six slices of 30 mm × 30 mm were cut from the pizza’s raised rim, then thirty-six measurements (6 slice × 6 sample) were performed for each typology of pizza base. The TPA test consisted of compressing the slice twice to 50% of its initial height, with a cross-head speed of 1 mm/s and a time of 10 s between compressions, using an aluminum probe plate (25 mm diameter) and a 50 N load cell.

### 2.9. Color Analysis of Cooked Pizza Base

The color analysis was performed by using an electronic eye IRIS Alpha-Mos (Visual Analyzer, IRIS VA 400, Alpha M.O.S., Toulouse, France). The results were shown according to the CIE L*, a*, b* scale. The parameters L* (brightness: 0 = black, 100 = white), a* (green (−), redness (+)), and b* (light blue (−), yellow (+)) were measured on the whole sample surface. Color differences (ΔE) were determined by using Equation (2) [20,21]:

(2) $$\Delta E=\sqrt{{\left(L-L_{0}\right)}^{2}+{\left(a-a_{0}\right)}^{2}+{\left(b-b_{0}\right)}^{2}}$$

where L_0_, a_0_, and b_0_ correspond to the CIE color parameters of the pizza control.

### 2.10. Statistical Analysis

The experimental data were expressed as mean ± SD (*n* = 6) and subjected to analysis of variance (ANOVA) by using the one-way analysis of variance procedures. The significant difference of means was analyzed by Duncan’s multiple range test, and *p* < 0.05 was considered to be statistically significant. JMP software 10.0 (SAS Institute, Cary, NC, USA) was used for data analysis.

## 3. Results and Discussion

The ZJP is a good source of the functional compounds largely proposed for food fortification [12]. The fortification of the Neapolitan pizza, the most consumed Italian traditional food in the world, represents an interesting strategy for promoting the functional benefits of ZJP to prevent diseases and improve human wellbeing.

The chemical composition and antioxidant properties of ZJP are shown in Table 1.

In agreement with the literature [13], the total sugars represent the most abundant constituents of ZJP. Among the total sugars, the insoluble dietary fibers (5.91 ± 0.12 g/100 g) were found to be much higher than soluble dietary fibers (1.64 ± 0.08 g/100 g). Insoluble fibers (cellulose, lignin, and hemicellulose) are known to have potential health benefits due to their ability to absorb water; this increases fecal mass and viscosity by promoting the movement of material through the digestive system [22]. The recommended amount of dietary fiber intake per adult is 25–38 g, and recent studies report that for every 10 g of additional fiber added to a diet, the mortality risk of coronary heart disease decreases by 17–35% [23,24]. The technological characteristics of dietary fibers are very interesting in food formulation as they can be responsible for texture change and the improvement of the stability of the food product during production and storage.

In addition, the regular intake of natural antioxidants such as phenols and flavonoids promotes the risk reduction of various diseases by counteracting oxidative stress. The phenol (17.62 ± 0.2 mg GAE/g) and flavonoid (3.51 ± 0.12 mg QE/g) contents of ZJP are higher compared to that detected in other products used for the food fortification [4,5]. Moreover, ZJP showed a significant DPPH^•^ and ABTS^•+^ radical scavenging capacity (Table 1). Thus, ZJP can be considered as a good fortifying agent suitable for the improvement of beneficial effects to health through its antioxidant and radical scavenging properties.

For this purpose, enriched pizza bases were prepared by adding ZJP at 2.5%, 5%, and 7.5% (*w*/*w*), and the results of phenol and flavonoid content measurements as well as the antioxidant ability detected by two free radical antioxidant methods (DPPH and ABTS) are reported in Table 2.

The phenol and flavonoid contents showed a positive association with the replacement of wheat flour by ZJP in the pizza base formulations (Control < ZJP 2.5% < ZJP 5.0% < ZJP 7.5%). As expected, a similar trend was observed for the antioxidant ability detected by DPPH and ABTS assays.

These results are attributed to the important amount of phytochemicals in the jujube fruit, particularly phenols (Table 1), which represent the main components with high antioxidant activity [11]. However, flavonoids and phenols can participate individually or synergistically in the antioxidant capacity [8]. Similar results were observed in the fortification of baked goods with natural raw materials, such as eggplant flour [6], jujube (var Lotus) powder [8], onion skin powder [18], mallow powder [25], and black cherry pomace extract [26], where the fortification provided better antioxidant abilities and with a linear relationship between TPC and antioxidant properties. Therefore, pizza bases fortified with ZJP had improved nutritional quality with better stability against oxidation.

The effects of the ZJP addition to the textural attributes of the fortified pizza bases were analyzed by using a texture profile analysis. The crust of the baked samples was compressed twice between the plates of the texture analyzer, which imitates the jaw action. The results show that the replacement of flour with ZJP significantly increases the hardness, gumminess, and chewiness (Table 3) with the following trend: Control < ZJP 2.5% < ZJP 5.0% < ZJP 7.5%. This behavior can be associated with the increase of insoluble dietary fiber due to the addition of ZJP (Table 1) and is in agreement with other studies in which the addition of fibers to the dough is able to increase the hardness and the derived parameters, such as chewiness and gumminess [8,18,27,28,29,30]. However, although these parameters showed a significant increase, the variation, in absolute value, was not high enough to modify the acceptability of the fortified products. In fact, the other direct attributes detected by the TPA, such as adhesiveness, springiness, and cohesiveness, showed non-significant differences between the fortified pizzas and the control.

Color is one of the main characteristics that defines the acceptability of food by consumers. To compare the effect of the ZJP addition on the Neapolitan pizza color, the total surface of samples was analyzed with an electronic eye, and the CIELab results obtained for all samples are presented in Table 4. The total color differences (ΔE) is an important parameter since it considers all differences encountered between the L*, a*, and b* values of the samples with respect to the control, giving a valid tool with which to evaluate the relationship between visual perception and the numerical analyses [31].

It is well-known that a ΔE value higher than one can be associated with a significant chromatic difference between the sample and the control. However, a ΔE < 2 can be noticed only by an experienced observer; for ΔE < 3.5, the difference can also be appreciated by an unexperienced observer; in contrast, a ΔE > 3.5 can produce a clear color difference between the samples [32]. Results reported in Table 4 indicate that a chromatic difference can be observed only in the samples containing 5% and 7.5% of ZJP, with a strong difference in the higher amount of ZJP. These results are principally associated with the reduction of L* and the increase of a* values (Table 4). The decrease in lightness is due to the higher fiber’s amount of ZJP, which, as reported by Najjaa et al. [8], is able to decrease a sponge cake’s lightness. Moreover, it is well-known that when a powder is added to the flour, its type and color may affect the chromatic perception of the final product, which can also be influenced by the baking process [33]. Thus, the significant increase (*p* < 0.05) of the a* value observed in the samples containing 5% and 7.5% of ZJP can be associated with the intrinsic color of ZJP, or to the colored compounds generated by caramelization and the Maillard reaction that occur during baking [34].

## 4. Conclusions

In conclusion, when ZJP is used as a fortifier in Neapolitan Pizza, the textural characteristics (hardness, gumminess, and chewiness) and the chromatic properties are affected as the amount of ZJP added is increased. However, the differences are not enough to change the overall acceptability of the products.

The incorporation of ZJP into Neapolitan pizza base formulation markedly increased the fiber, total phenolic and flavonoid contents, and the radical scavenging activity. Therefore, ZJP could be considered as a potential, health-promoting, functional ingredient without promoting negative effects and without changing the desirable physical and sensorial characteristics of the Neapolitan pizza, although a sensory analysis study is necessary to assess the consumer response and satisfaction. Further studies are needed to verify its health-giving properties in vivo, after ingestion and full digestion.

## Figures and Tables

**Table 1:** Proximate composition and antioxidant properties of ZJP.

Components	Value
Total carbohydrates (g/100 g DW)	81.46 ± 0.34
Soluble dietary fibers (g/100 g DW)	1.64 ± 0.08
Insoluble dietary fibers (g/100 g DW)	5.91 ± 0.12
Fat (g/100 g DW)	3.44 ± 0.09
Proteins (g/100 g DW)	6.83 ± 0.13
Moisture (g/100 g DW)	4.58 ± 0.18
Ash (g/100 g DW)	3.29 ± 0.09
Phenols (mg GAE/g DW)	17.62 ± 0.02
Flavonoids (mg QE/g DW)	3.51 ± 0.12
ABTS (radical scavenging activity %)	61.07 ± 1.42
DPPH (radical scavenging activity %)	50.05 ± 2.31

Each value is expressed as mean ± SD (*n* = 6).

**Table 2:** Total phenolic content (TPC), total flavonoid content (TFC), and radical scavenging activity of pizza base enriched with ZJP, tested by ABTS^•+^ and DPPH^•^ assays.

Samples	TPC (mg GAE/g DW)	TFC (mg QE/g DW)	ABTS (%)	DPPH (%)
Control	0.82 ± 0.04 ^a^	0.01 ± 0.01 ^a^	33.18 ±1.33 ^a^	18.46 ± 0.70 ^a^
ZJP 2.5%	1.02 ± 0.07 ^b^	0.06 ± 0.01 ^b^	50.69 ± 3.08 ^b^	23.57 ± 0.37 ^b^
ZJP 5.0%	1.28 ± 0.09 ^c^	0.09 ± 0.01 ^c^	65.86 ± 2.77 ^c^	45.46 ± 0.26 ^c^
ZJP 7.5%	1.51 ± 0.05 ^d^	0.11 ± 0.03 ^c^	78.52 ± 3.74 ^d^	55.29 ± 0.51 ^d^

Each value is expressed as mean ± SD (*n* = 6). Means with the same letters in the same column are not significantly different (*p* < 0.05), according to Duncan’s multiple range test.

**Table 3:** Effect of ZJP enrichment on the textural profile of pizza base variants.

Samples	Hardness (N)	Adhesiveness (Nmm)	Cohesiveness	Springiness (mm)	Gumminess (N)	Chewiness (mJ)
Control	3.75 ± 0.06 ^a^	0.27 ± 0.02 ^a^	0.72 ± 0.01 ^a^	9.58 ± 0.20 ^a^	2.69 ± 0.01 ^a^	25.81 ± 0.71 ^a^
ZJP 2.5%	4.31 ± 0.28 ^b^	0.25 ± 0.03 ^a^	0.71 ± 0.01 ^a^	9.72 ± 0.27 ^a^	3.08 ± 0.16 ^b^	30.12 ± 0.99 ^b^
ZJP 5.0%	5.00 ± 0.28 ^c^	0.24 ± 0.02 ^a^	0.70 ± 0.02 ^a^	9.81 ± 0.03 ^a^	3.46 ± 0.10 ^c^	33.81 ± 0.68 ^c^
ZJP 7.5%	5.82 ± 0.17 ^d^	0.20 ± 0.02 ^b^	0.70 ± 0.03 ^a^	9.96 ± 1.27 ^a^	4.08 ± 0.21 ^d^	40.75 ± 2.31 ^d^

Each value is expressed as mean ± SD (*n* = 36). Means with same letters in the same column are not significantly different (*p* < 0.05), according to Duncan’s multiple range test.

**Table 4:** Color values of pizza base variant.

Samples	L*	a*	b*	∆E
Control	62.77 ± 0.37 ^a^	1.14 ± 0.24 ^a^	27.90 ± 0.21 ^a^	-
ZJP 2.5%	62.50 ± 0.67 ^a^	1.39 ± 0.03 ^a^	27.70 ± 0.50 ^a^	0.41
ZJP 5.0%	60.18 ± 0.58 ^b,c^	1.69 ± 0.03 ^a,b^	27.61 ± 0.07 ^a^	2.66
ZJP 7.5%	58.51 ± 0.64 ^c^	2.19 ± 0.15 ^b^	28.23 ± 0.55 ^a^	4.40

Each value is expressed as mean ± SD (*n* = 6). Means with same letters in the same column are not significantly different (*p* < 0.05), according to Duncan’s multiple range test.
